# Supplementary material for: Evaluation of Pharmacokinetic and Toxicological Parameters of Arnica Tincture after Dermal Application In Vivo
Source: Pharmaceutics. 2022 Nov 4;14(11):2379. doi: 10.3390/pharmaceutics14112379 (PMC9695956; doi:10.3390/pharmaceutics14112379)
Supplement: Supplementary file 1 [file pharmaceutics-14-02379-s001.zip › pharmaceutics-1980906-supplementary.pdf]

pharmaceutics

Supplementary Materials: Evaluation of Pharmacokinetic and Toxicological Parameters of Arnica Tincture after Dermal Application In Vivo

Franziska M. Jürgens, Sara M. Robledo and Thomas J. Schmidt

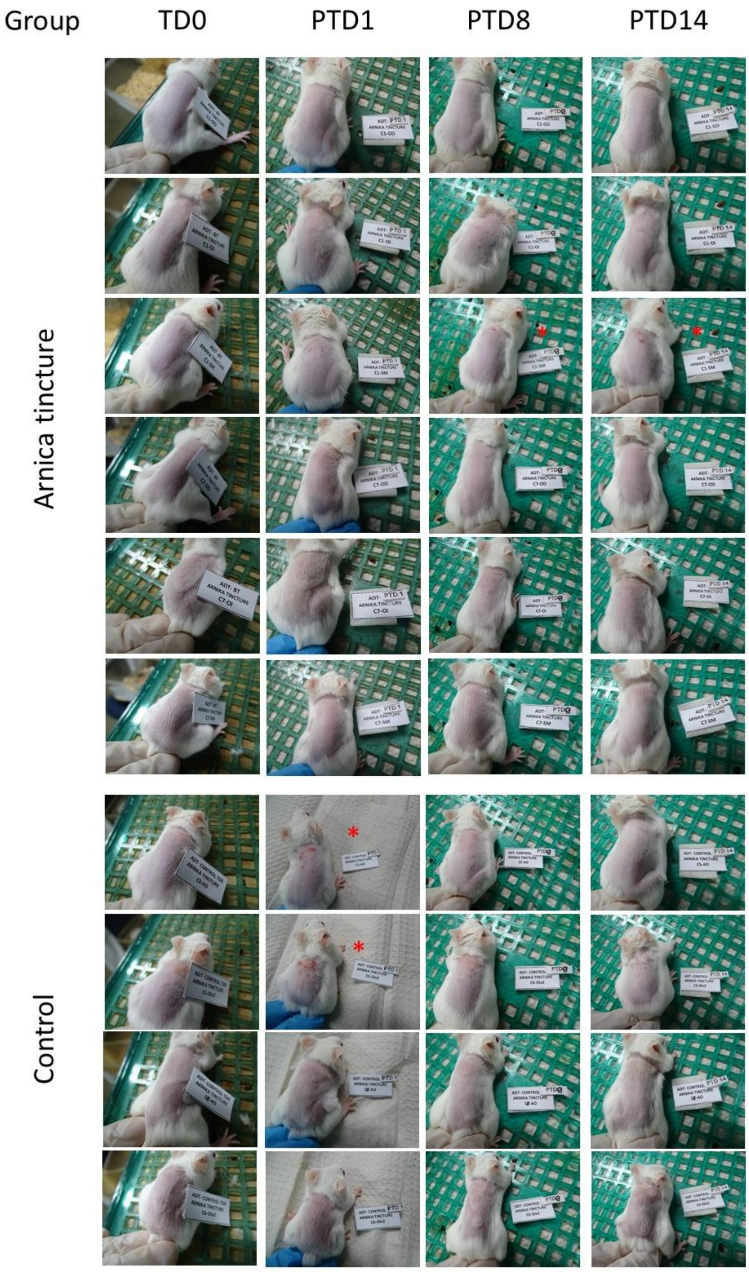

Figure S1. Appearance of skin area exposed to the tincture in comparison to untreated mice (acute dermal toxicity study). *Mice with erythematous lesions.

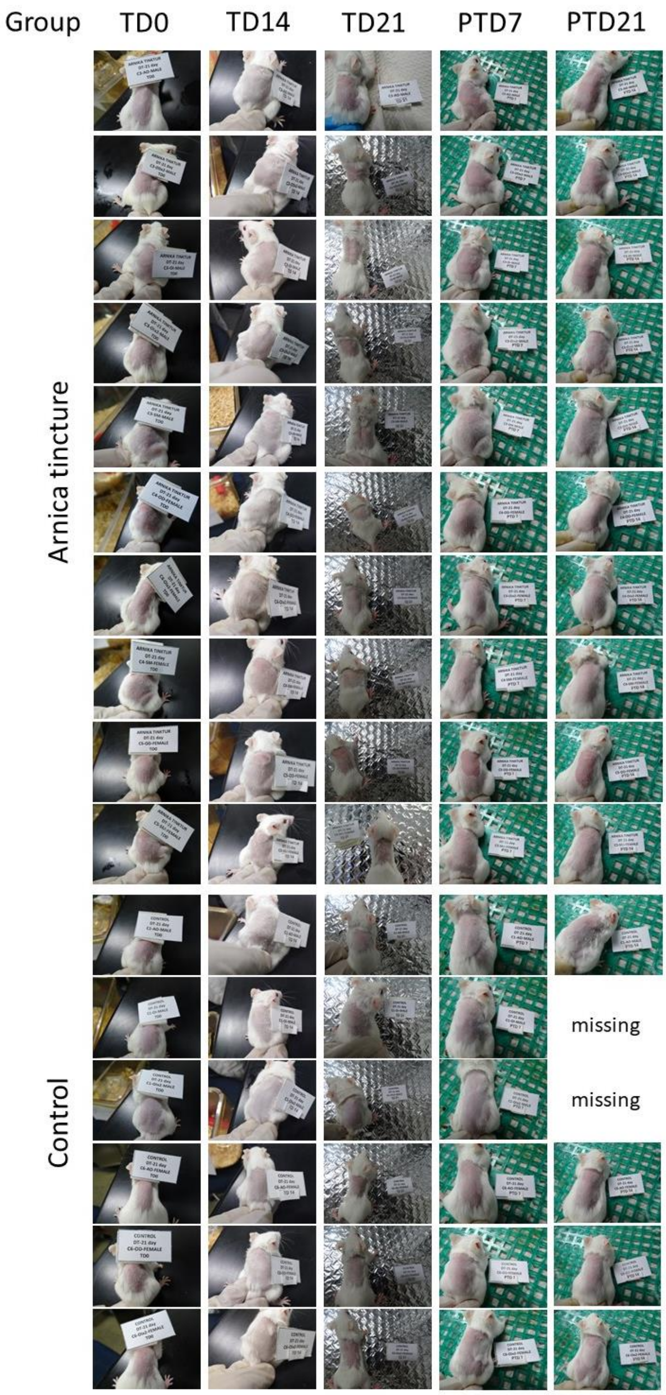

Figure S2. Appearance of skin area exposed to the tincture in comparison to untreated mice (repeated dose dermal toxicity study).
